# Metabolomics-Based Study on the Anticonvulsant Mechanism of *Acorus tatarinowii*: GABA Transaminase Inhibition Alleviates PTZ-Induced Epilepsy in Rats

**DOI:** 10.3390/metabo15030175

**Published:** 2025-03-04

**Authors:** Liang Chen, Jiaxin Li, Chengwei Fang, Jiepeng Wang

**Affiliations:** 1 School of Pharmacy, Guizhou University of Traditional Chinese Medicine, Guiyang 550025, China; chenliang029@gzy.edu.cn (L.C.); lijiaxin048@gzy.edu.cn (J.L.); maxiuping486@gzy.edu.cn (C.F.); 2 School of Basic Medical Sciences, Hebei University of Chinese Medicine, Shijiazhuang 050200, China

**Keywords:** epilepsy, *Acorus tatarinowii* Schott, metabolomics, plasma, Gamma-aminobutyrate transaminase

## Abstract

Background/Objectives: Epilepsy is a common chronic and recurrent neurological disorder that poses a threat to human health, and *Acorus tatarinowii* Schott (ATS), a traditional Chinese medicine, is used to treat it. This study aimed to determine its effects on plasma metabolites. Moreover, the possible mechanism of its intervention in epilepsy was preliminarily explored, combined with network pharmacology. Methods: An epileptic model of rats was established using pentylenetetrazol. The potential targets and pathways of ATS were predicted by network pharmacology. Ultra Performance Liquid Chromatography–Quadrupole–Time of Flight Mass Spectrometrynce Liquid Chromatography–Quadrupole–Time of Flight Mass Spectrometryance Liquid Chromatography–Quadrupole–Time of Flight Mass Spectrometry and statistical analyses were used to profile plasma metabolites and identify ATS’s effects on epilepsy. Results: Kyoto Encyclopedia of Genes and Genomes enrichment analysis revealed that ATS was involved in regulating multiple signaling pathways, mainly including the neuroactive ligand–receptor interaction and GABAerGamma-aminobutyrate transaminaseAminobutyrate Transaminaseapse signaling pathway. ATS treatment restored 19 metabolites in epiGamma-aminobutyrate transaminaseminobutyrate Transaminase rats, affecting lysine, histidine, and purine metabolism. GABA-T was found as a new key target for treating epilepsy with ATS. The IC_50_ of ATS for inhibiting GABA-T activity was 108.9 μg/mL. Through metabolomic analysis, we detected changes in the levels of certain metabolites related to the GABAergic system. These metabolite changes can be correlated with the targets and pathways predicted by network pharmacology. One of the limitations of this study is that the correlation analysis between altered metabolites and seizure severity remains unfinished, which restricts a more in-depth exploration of the underlying biological mechanisms. In the future, our research will focus on conducting a more in-depth exploration of the correlation analysis between altered metabolites and seizure severity. Conclusions: These results improved our understanding of epilepsy and ATS treatment, potentially leading to better therapies. The identification of key metabolites and their associated pathways in this study offers potential novel therapeutic targets for epilepsy. By modulating these metabolites, future therapies could be designed to better manage the disorder. Moreover, the insights from network pharmacology can guide the development of more effective antiepileptic drugs, paving the way for improved clinical outcomes for patients.

## 1. Introduction

Epilepsy, a common chronic neurological disorder, is characterized by recurrent, unprovoked seizures due to abnormal brain electrical activity. It is classified as focal-onset and generalized seizures, each with distinct symptoms. Current treatments mainly include antiepileptic drugs like carbamazepine and valproic acid, along with surgical and neuromodulation options for drug-resistant cases. However, many patients still face seizure control issues [1]. Its pathogenesis is multifaceted, with seizure activity impacting both the structural and functional aspects of brain circuits, thereby contributing to seizure progression [2]. Status epilepticus can trigger neurodegeneration, significantly impairing the patient’s quality of life [3]. Despite the availability of at least 25 antiepileptic drugs, approximately 30% of epilepsy cases exhibit resistance to drug therapy [4]. Consequently, many individuals seek alternative or complementary therapies due to inadequate seizure control.

With a long history of use, traditional Chinese medicine (TCM) is increasingly recognized as a novel approach for epilepsy treatment [5]. Herbal combinations have demonstrated effectiveness by targeting multiple mechanisms, including antioxidative, anti-inflammatory, enhanced GABAergic effects, modulation of NMDA receptors, and neuroprotection. This growing interest in TCM for epilepsy is reflected in recent research [6,7].

*Acorus tatarinowii* Schott (ATS), a key component in ancient Chinese medicine, has been used for over 2000 years, as documented in Shennong’s *Classic of Materia Medica* from the Han Dynasty. It is a perennial herbaceous plant belonging to the family Araceae. This plant contains various bioactive ingredients, among which volatile oils are the main ones, including α-pinene, β-pinene, camphor, as well as α-asarone, β-asarone and other components. Additionally, it also contains flavonoids, alkaloids, phenylpropanoids, steroids, and other substances [8,9]. Its role in treating neurological disorders like epilepsy, Alzheimer’s, insomnia, and depression has been highlighted [10,11]. The volatile oil of ATS is considered its primary pharmacologically active compound [12]. Despite extensive research on its chemistry and effects, the in vivo mechanism of ATS remains largely unexplored. TCM employs a complex, multi-component approach to treat diseases, making it challenging to analyze using conventional methods.

Metabolomics, a key part of systems biology, studies changes in small-molecule metabolites after stimuli or interventions, identifying differences and explaining biological processes [13,14]. Aligned with TCM’s holistic approach, metabolomics shows promise in evaluating treatment effectiveness and elucidating biochemical mechanisms [15,16]. This study aimed to reveal ATS’s mechanisms in epilepsy treatment using untargeted plasma metabolomics, combined with network pharmacology.

## 2. Materials and Methods

### 2.1. Chemicals

Methanol and acetonitrile were acquired from Concord Technology (Tianjin, China). Pentylenetetrazol (PTZ) was sourced from Aladdin Co., Ltd. (Shanghai, China, F2218455). Other analytical-grade chemicals and doubly distilled water were used throughout the study.

### 2.2. Network Pharmacology Profiling

Chemical ingredients and the targets of ATS were obtained from the TCMSP (https://old.tcmsp-e.com/tcmspsearch.php (accessed on 2 March 2022)) and BATMAN-TCM (http://bionet.ncpsb.org/batman-tcm/ (accessed on 25 March 2022)) databases. The targets of active ingredients were predicted through the Swiss TargetPrediction database. Using “possibility” > 0 as the screening criterion, duplicate items were merged and removed to obtain the action targets. The interaction network was visualized using Cytoscape 3.7.1 software. Enrichment analyses for Kyoto Encyclopedia of Genes and Genomes (KEGG) and Gene Ontology (GO) were performed in the DAVID database (Frederick, MD, USA), and results were visualized using the Bioinformatics online tool (http://www.bioinformatics.com.cn (accessed on 6 May 2022)). When performing KEGG pathway enrichment, pathways with *p* < 0.01 were further analyzed.

### 2.3. Extraction of Essential Oil from ATS

ATS, acquired from Kangmei Pharmaceutical Co., Ltd. (Shenzhen, China, 210702451), was accurately weighed and soaked in nine times its weight of distilled water for four hours. Subsequently, it was subjected to steam distillation for six hours to yield a thick, yellow oil-like substance, i.e., volatile oil. The volatile oil from ATS was dehydrated using anhydrous sodium sulfate and stored under sealed conditions at 4 °C for future use. The extraction rate of volatile oil from ATS was determined to be 1.68%.

### 2.4. Animals and Ethical Approval

Male Sprague Dawley rats weighing 200 ± 20 g were purchased from Tianqin Biotechnology Co., Ltd. (Changsha, China). They were housed in a specific-pathogen-free (SPF) facility under controlled conditions of temperature (22 ± 2 °C), relative humidity (55 ± 5%), and a 12 h/12 h light/dark cycle. The experiment followed the National Guidelines for the Use of Experimental Animals for Health Care, with approval from the Institutional Animal Ethics Committee of Guizhou University of Traditional Chinese Medicine, China.

### 2.5. Experimental Design

Before the experiment, the animals were acclimatized at the research center for 7 days or more. Seizures were induced in SD rats by intraperitoneal injection of PTZ in saline solution (35 mg/kg body weight) every other day for 30 days. Male rats in the control group (*n* = 6) received an equivalent volume of saline solution. Animal behavior during seizures was monitored using the Racine scale with minor modifications as previously described [17,18]: Stage 1, facial clonus, staring, and wet-dog shakes. Stage 2, vertical movement of the head. Stage 3, rhythmic contraction of forelimb. Stage 4, rhythmic contraction along with upright raising of forelimb. Stage 5, rearing, jumping, falling, and recurrent seizure. The model was considered successful if rats experienced more than three consecutive episodes of Racine V (forelimb clonus, rhythmic nodding, facial clonus, hind limb standing, and falling). The epileptic rats were randomly divided into the model group (*n* = 6) or ATS group (*n* = 6) (epilepsy was induced in rats with PTZ and treated with ATS at a dose of 50 mg/kg once daily for four weeks).

### 2.6. Sample Collection

After a 12 h fast, blood was collected from rat abdominal aortas using EDTA anticoagulant tubes. The samples were centrifuged at 3000 rpm for 10 min in a 4 °C centrifuge, and the supernatant was stored in cryopreserved tubes at −80 °C for further analysis.

### 2.7. Sample Preparation

The plasma samples were processed by firstly combining them with 500 µL of extract solvent (methanol/acetonitrile = 1:1) using a vortex mixer for 30 s, followed by 10 min of ultrasonication in an ice water bath. After incubating the mixture at −20 °C for 1 h, it was centrifuged at 4 °C and 12,000 rpm for 15 min. The resulting supernatant (500 µL) was transferred to an EP tube and dried in a vacuum concentrator. Subsequently, 160 µL of extract solvent (acetonitrile/water = 1:1) was added to the dried sample, vortexed, and centrifuged using the same method. The resulting supernatant was analyzed by UPLC–Q-TOF-MS.

For method validation, 10 µL of each plasma sample was combined to create quality control (QC) samples.

### 2.8. UPLC Conditions

Metabolomics analysis was performed using a UPLC–MS system consisting of a Waters Acquity I-Class PLUS ultra-high performance liquid chromatography coupled with a Waters Xevo G2-XS QTof high-resolution mass spectrometer. HPLC analysis was conducted using an Acquity UPLC HSS T3 chromatographic column (2.1 mm × 100 mm, 1.8 µm, Waters, Milford, MA, USA). The flow rate was 0.4 mL/min, and the injection volume was 1 µL. The mobile phase was a mixture of 0.1% formic acid (A) and acetonitrile (B). Elution conditions were set as follows: 0–0.25 min, 2% B; 0.25–10 min, 2–98% B; 10–13 min, 98% B; 13–13.1 min, 98–2% B; and 13.1–15 min, 2% B.

### 2.9. Mass Spectrometry Conditions

The Waters Xevo G2-XS QTOF high-resolution mass spectrometer was used for mass spectrometry analysis. Detection was carried out in both positive and negative electrospray ionization modes. Scans were conducted over the m/z range of 50–1200. The ESI ion source parameters were configured as follows: ion source temperature, 100 °C; cone-hole voltage, 30 V; capillary voltage, 2500 V (positive ionization) or −2000 V (negative ionization).

### 2.10. Inhibition Study of GABA-Transaminase

The activity of GABA-transaminase (GABA-T) was determined under incubation conditions of 37 °C for 30 min. The reaction was initiated by adding substrate GABA solution and terminated in an ice bath. The solution without GABA was used to initiate the enzyme-catalyzed reaction as the control. The absorbance value of nicotinamide adenine dinucleotide (NADH) was detected at 340 nm using a UV spectrophotometer, and the activity of GABA-T was reflected by the amount of NADH produced during the incubation reaction.

### 2.11. Statistical Analysis

The initial data obtained with MassLynx V4.2 (Waters, Milford, MA, USA) were analyzed using Progenesis Qi software 2.3 (Waters, Milford, MA, USA), involving alignment, peak extraction, and normalization. Orthogonal projections to latent structures-discriminant analysis (OPLS-DA) and principal component analysis (PCA) were conducted using ropls 1.6.2 and prcomp (R base function) 3.6.1, respectively. Differential metabolites were identified based on variable importance in projection (VIP) ≥ 1 and *p* values < 0.05 using a *t*-test.

## 3. Results

### 3.1. Network Pharmacology Analysis

**Figure 1:**
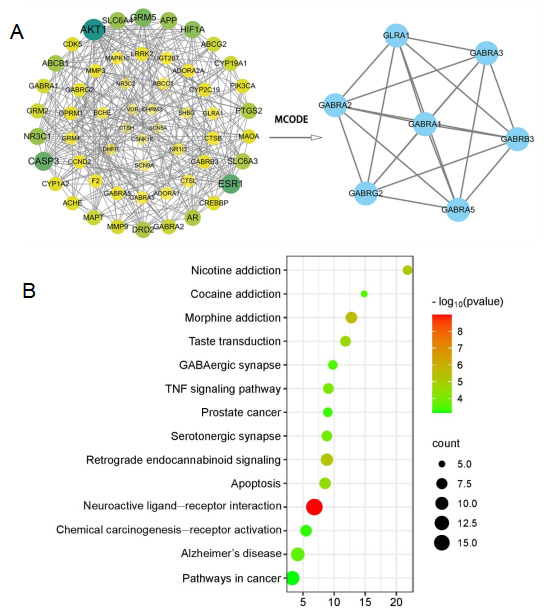
Network pharmacology analysis. (**A**) PPI network and topological analysis diagram of target points for treating epilepsy with ATS; (**B**) bubble plot of KEGG enrichment analysis.

In short, we imported the intersection targets of ATS and epilepsy into a String database to obtain the interaction relationships between target proteins, and used Cytoscape software 3.9.1 to draw a protein–protein interaction network (PPI). Further analysis of sub network modules was conducted using the MCODE plugin in Cytoscape software 3.9.1 to screen for key genes involved in the antiepileptic effects of ATS, including gamma aminobutyric acid type A receptor genes (GABRA1, GABRA2, GABRA3, and GABRA5), gamma aminobutyric acid type A receptor beta 3 subunit (GABRB3), gamma aminobutyric acid type A receptor gamma 2 subunit (GABRG2), and glycine receptor alpha 1 subunit (GLRA1) (Figure 1A). On the other hand, KEGG analysis revealed pathways such as the neuroactive ligand–receptor interaction and GABAergic synapse (Figure 1B).

### 3.2. QC Analysis

**Figure 2:**
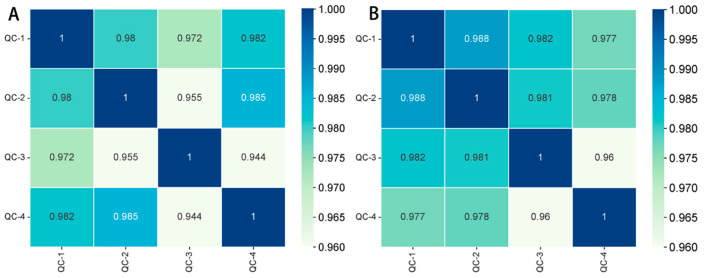
Pearson correlation analysis of QC samples. (**A**) Correlation between positive QC samples; (**B**) correlation between negative QC samples.

Plasma metabolomic profiles were generated using UPLC–MS analysis in both negative and positive ion modes. To ensure accuracy and stability, one QC sample was analyzed for every six samples due to the susceptibility of metabolism to external influences and its rapid fluctuations. Additionally, Pearson correlation coefficients were computed for the QC samples based on the relative quantitative values of metabolites. A correlation coefficient close to 1 indicates superior overall test stability and higher data quality [19]. In this study, the correlation coefficient approached 1 in both negative and positive ion modes, indicative of good data stability and high quality, thereby providing reliable results (Figure 2).

### 3.3. Metabolomics Analysis

**Figure 3:**
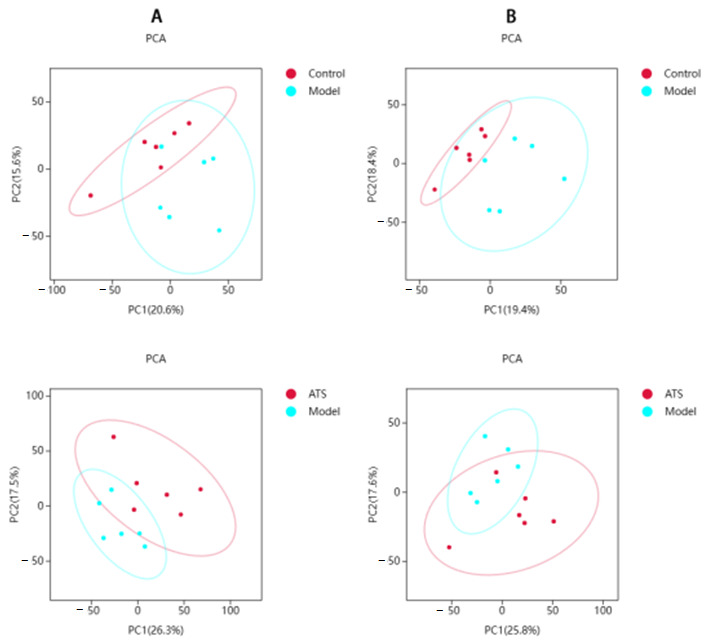
PCA score plots in different ion modes. (**A**) Positive PCA score plots and (**B**) negative PCA score plots.

**Figure 4:**
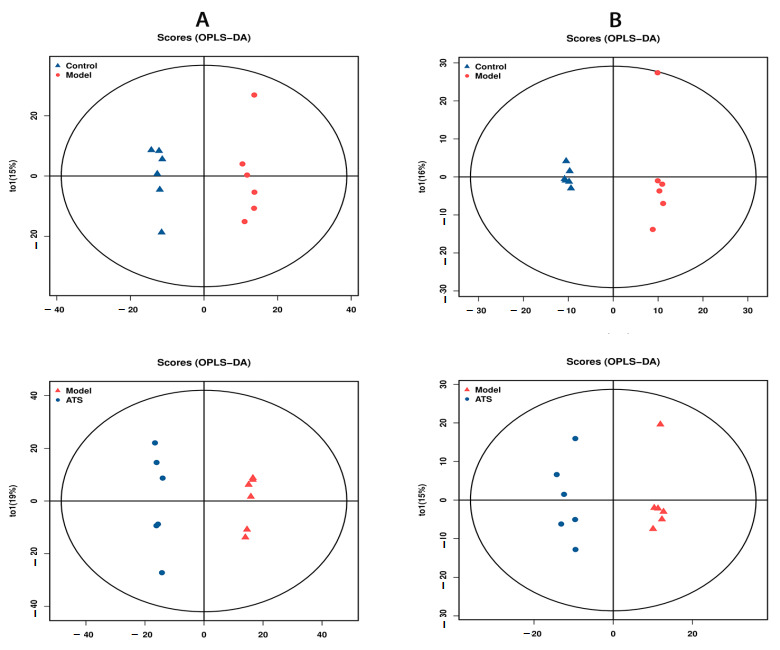
OPLS-DA score plots in different ion modes. (**A**) Positive OPLS-DA score plots and (**B**) negative OPLS-DA score plots.

**Figure 5:**
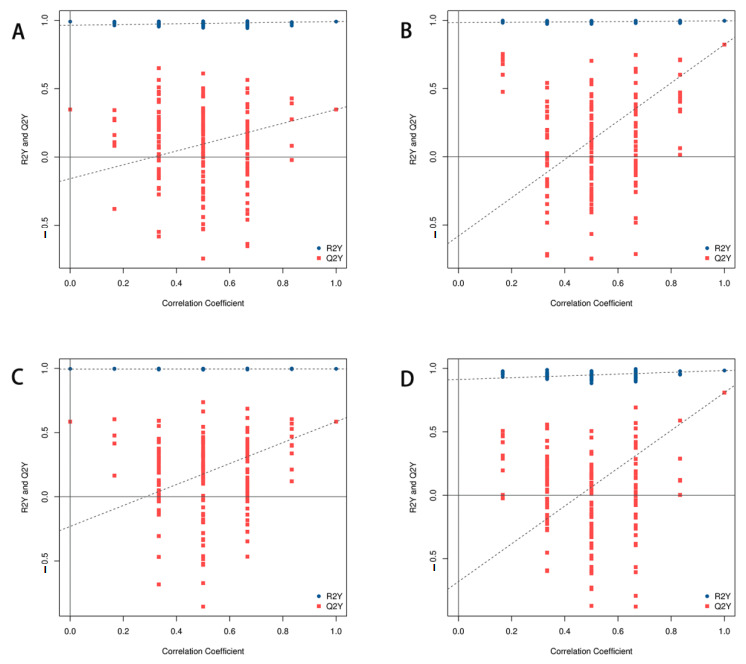
Permutation test results of OPLS-DA model. (**A**) Positive scatter plots for control group vs. model group; (**B**) positive scatter plots for model group vs. ATS group; (**C**) negative scatter plots for control group vs. model group; and (**D**) negative scatter plots for model group vs. ATS group.

Unsupervised PCA was performed to find clustering trends of metabolic profiles among groups (Figure 3). The results revealed that in both negative and positive ion modes, the model group and the control group clustered well on the PCA score map. To improve the separation of metabolic profiles, a supervised OPLS-DA model was applied following the PCA outcomes. The results revealed disparities in the plasma levels of endogenous metabolites in both positive and negative ion modes, as illustrated in Figure 4. To evaluate overfitting risk, 200 random permutation tests were executed on the model (Figure 5). The significant differences in plasma samples were observed between the control and model groups (Q2Y = −0.186 for positive ion mode and Q2Y = −0.247 for negative ion mode). The Q2 regression line’s negative intercept suggested the model’s absence of overfitting and its high reliability.

### 3.4. Identification of Differentially Expressed Metabolites

**Figure 6:**
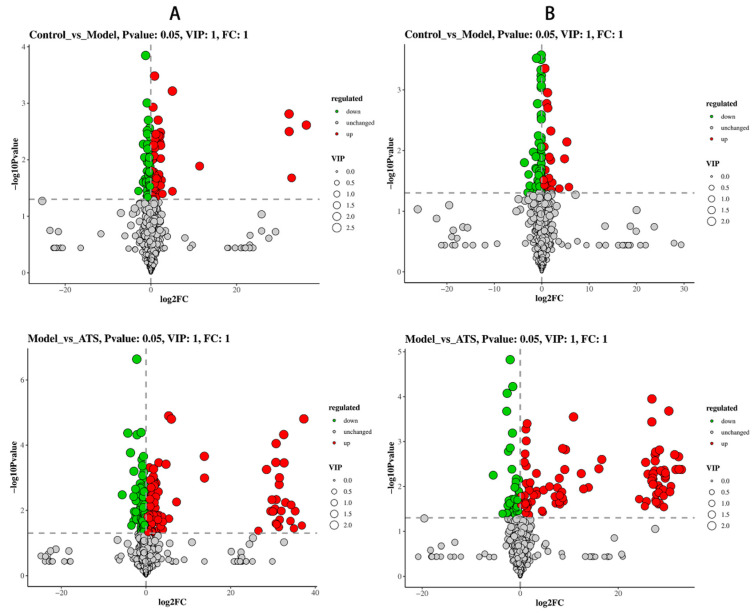
Volcano plots of differential metabolites in the OPLS-DA model. (**A**) Positive volcano plot and (**B**) negative volcano plot.

Utilizing the OPLS-DA model, variables with VIP scores ≥ 1 were selected, and a *t*-test was applied to detect significant group differences (*p* < 0.05). Differential metabolites identified in the OPLS-DA model were visualized and filtered using a volcano plot (Figure 6). A total of 200 metabolites were significantly expressed between the control and model groups in both positive and negative ion modes. In the model group, 74 metabolites were up-regulated and 126 were down-regulated compared to the control group. Among these potential biomarkers, 19 metabolites (e.g., D-erythro-1-(Imidazol-4-yl)glycerol 3-phosphate, indolelactic acid) were significantly restored to control levels by ATS intervention (Table 1). These results indicated that modulating the levels of these differentially expressed metabolites may effectively alleviate seizures.

### 3.5. Analysis of Metabolic Pathways of Potential Biomarkers

**Figure 7:**
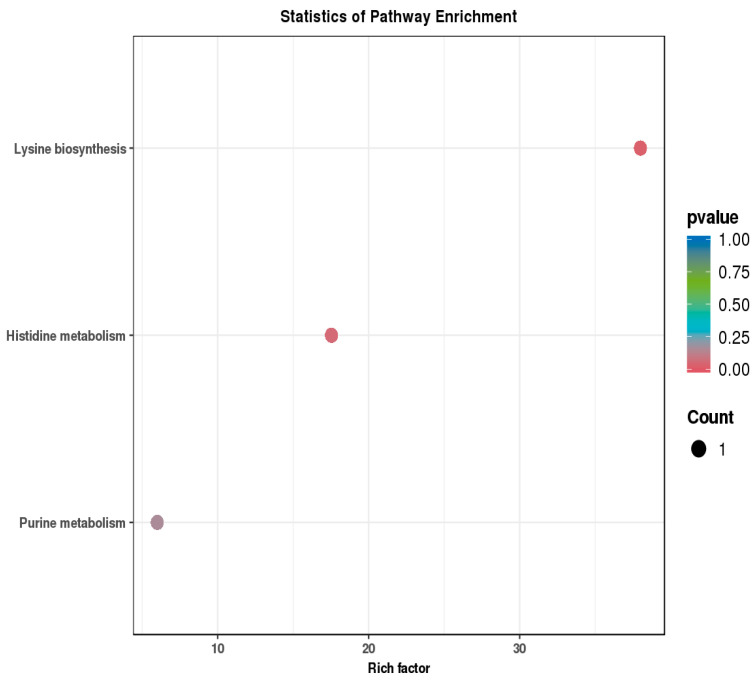
Metabolic pathway enrichment analysis of differential metabolites.

The 19 differential metabolites were subjected to pathway enrichment analysis, leading to the creation of a metabolic pathway map using the Kyoto Encyclopedia of Genes and Genomes (KEGG), a freely available web-based database resource [20]. Figure 7 illustrates the KEGG enrichment analysis diagram of these differential metabolites. This analysis identified three key metabolic pathways: lysine biosynthesis, histidine metabolism, and purine metabolism. Lysine, which serves as a signaling molecule, plays a crucial role in regulating translation initiation factors, thereby exerting an impact on protein biosynthesis. When lysine metabolism is impaired, it gives rise to an increase in the levels of glutarate in the brain. This elevated glutarate then inhibits glutamate decarboxylase, which in turn reduces the synthesis of GABA. The reduction in GABA synthesis leads to severe neurological deficits, and eventually culminates in epilepsy. Purine metabolites play a significant role in epilepsy. Imbalances in purine metabolism can disrupt the energy supply and neurotransmitter regulation in neurons, leading to abnormal electrical activities in the brain and increasing the susceptibility to epileptic seizures. Histidine metabolism is closely linked to epilepsy. Abnormalities in histidine metabolism can disrupt the production of histamine, a neurotransmitter-like substance. Since histamine is involved in modulating the brain’s seizure threshold, such disruptions may increase the likelihood of epileptic seizures. These findings indicated that ATS may primarily influence epilepsy treatment by targeting these metabolic pathways.

### 3.6. Inhibition Study of GABA-T

**Figure 8:**
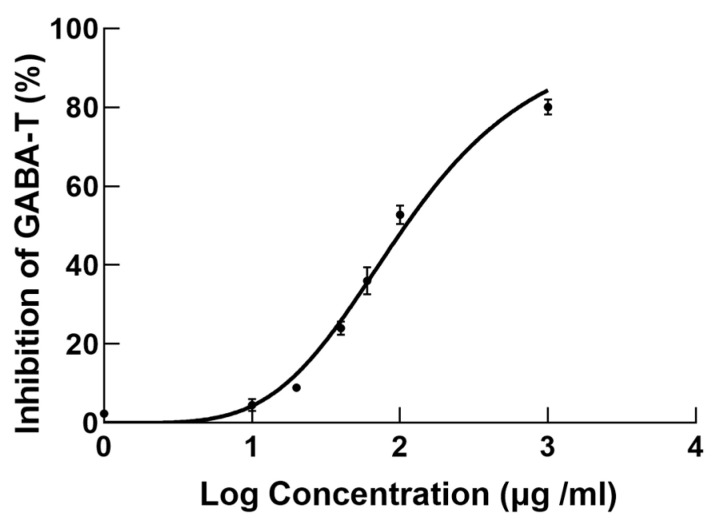
Inhibition curve of ATS on GABA-T (*n* = 3). Error bars indicate the standard error of the mean (SEM).

ATS had a concentration-dependent inhibitory effect on GABA-T activity, as shown in Figure 8. The IC50 of ATS for inhibiting GABA-T activity was 108.9 μg/mL, which was lower than the reported value of vigabatrin (600 μg/mL) in the literature [21]. The results of the enzyme inhibition experiments indicated that the antiepileptic effect of ATS may be related to GABA-T, laying the foundation for further elucidating the antiepileptic mechanism of ATS.

## 4. Discussion

Previous reports have shown that both decoction of and essential oil extraction from ATS have antiepileptic effects [22]. However, the mechanism of the antiepileptic effect of the volatile oil from ATS has not been elucidated. In the research of network pharmacology, KEGG enrichment analysis results showed that ATS was involved in regulating multiple signaling pathways, mainly including the neuroactive ligand–receptor interaction and GABAergic synapse signaling pathway. The neuroactive ligand–receptor interaction signaling pathway is a collection of receptors and ligands related to intracellular and extracellular signaling pathways in the plasma membrane center [23]. After binding to the corresponding receptors, they can participate in the regulation of biological functions such as physiological rhythms, emotions, learning, and memory in the body [24]. GABA is a neurotransmitter that can inhibit neuronal excitation. A decrease in the brain GABA level is closely related with several neurologic disorders including epilepsy, Parkinson’s disease, Huntington’s chorea, and Alzheimer’s disease [25,26]. Inhibition of GABA-T will increase the concentration of GABA in the brain and therefore should be useful for treating pathological conditions associated with low brain GABA levels [27].

Metabolic disruptions are closely tied to epilepsy, as even subtle changes post-seizure can alter metabolite levels. To understand how ATS combats epilepsy, plasma metabolomics via UPLC–Q-TOF-MS was used. Analysis showed clear separation among control, model, and ATS groups. ATS significantly restored 19 metabolites. Pathway analysis linked this restoration to lysine biosynthesis, histidine metabolism, and purine metabolism, which are key to ATS’s antiepileptic effects.

Epileptic seizures are triggered by the neuroendocrine system, which releases hormones. Lysine, aside from being a precursor for protein synthesis, also acts as a regulator of endocrine hormone release, facilitating the synthesis and secretion of insulin-like growth factor, insulin, and growth hormone [28]. Functioning as a signaling molecule, lysine regulates translation initiation factors, influencing protein biosynthesis. Impairment of lysine metabolism results in elevated brain glutarate levels, inhibiting glutamate decarboxylase, reducing γ-aminobutyric acid (GABA) synthesis, and leading to severe neurological deficits, ultimately causing epilepsy [29,30]. Both L-lysine and D-lysine significantly prolong the latency of epileptic seizures and exert antiepileptic effects via GABAergic transmission. The study noted a substantial increase in plasma metabolite (2R, 3R)-3-Methylotrophinyl-N6-lysine in model rats, indicating heightened lysine metabolism and metabolite formation. ATS treatment notably lowered this metabolite, implying a link between the lysine biosynthesis pathway and ATS’s therapeutic effects on epilepsy.

Histamine, a crucial neurotransmitter in the brain, plays diverse neuroregulatory roles. Reduced brain histamine levels can potentially trigger conditions like schizophrenia, epilepsy, and insomnia. Studies indicate that intracerebral histidine administration reduces alginate-induced seizures in mice [31]. Carnosine, a histidine precursor, can prevent PTZ-induced seizures during provocation and aggression in rats [32]. In this study, plasma levels of the metabolite D-erythro-1-(Imidazol-4-yl)glycerol 3-phosphate were significantly elevated in epileptic rats, with enrichment in the histidine metabolism pathway. The decrease in D-erythro-1-(Imidazol-4-yl)glycerol 3-phosphate levels following ATS essential oil administration suggests that ATS may regulate seizures through the histidine metabolism pathway.

Purine metabolites are fundamental for DNA and RNA, providing cofactors and energy for cell survival and growth. Adenosine and adenine nucleotides act as signaling molecules, activating purinergic receptors P1 and P2. While A1 adenosine receptor activation is anticonvulsant, A2A receptor activation may induce seizures. ATP, adenosine, and purine are key in epilepsy. In this study, plasma adenine levels were higher in model rats than controls, and ATS treatment reduced these levels, indicating purine metabolism’s role in epilepsy regulation.

Regarding the relationship between network pharmacology and metabolomics, network pharmacology focuses on the complex network of drug–target–disease relationships, aiming to explore the multi-target and multi-pathway mechanisms of drugs. Metabolomics, on the other hand, analyzes the small molecule metabolites in biological systems, providing insights into the metabolic status and physiological functions. In our research on ATS, there is an inherent connection between them. By integrating network pharmacology and metabolomics, we identified the key metabolites corresponding to the targets predicted by network pharmacology. These metabolites can serve as biomarkers to further verify the predicted targets and pathways. For example, through metabolomic analysis, we detected the changes in the levels of certain metabolites related to the GABAergic system. These metabolite changes can be correlated with the targets and pathways predicted by network pharmacology, thus establishing a connection between the two.

Lysine is one of the essential amino acids for humans and mammals. Blocking the metabolism of lysine, hydroxylysine, and tryptophan, inhibiting GABA-T, and reducing GABA synthesis may be important factors leading to epilepsy. Studies have shown that L-lysine and D-lysine significantly prolong the latency of epileptic seizures, exerting their anticonvulsant activity through GABAergic transmission. Both the plasma metabolomics and network pharmacology results indicated that the antiepileptic effect of ATS was closely related to the neurotransmitter GABA. Therefore, we determined the inhibitory effect of ATS on GABA-T in the validation experiment. GABA is deaminated to succinic semialdehyde by GABA-T. Increasing the concentration of GABA in the brain can enhance the neural inhibitory effect of the GABAergic system, thereby achieving the goal of preventing epilepsy. The inhibitory effect of ATS on GABA-T was dose-dependent. GABA-T may be a newly discovered key target for treating epilepsy with ATS. In our study, based on network pharmacology analysis, we found that some active ingredients of ATS may act on key targets in the GABAergic pathway. Through in vitro experiments, we further verified that ATS can regulate the activity of relevant enzymes in the GABAergic pathway, thereby affecting the synthesis, release, and re-uptake of GABA. This regulation ultimately leads to the modulation of the excitability of the central nervous system, which is consistent with the sedative and hypnotic effects of ATS.

## 5. Conclusions

In conclusion, our study shows that ATS administered intragastrically causes notable metabolic shifts in the plasma of epileptic rats. Metabolomics analysis showed that ATS treatment restored the levels of 19 metabolites in epileptic rats, primarily related to lysine biosynthesis, histidine metabolism, and purine metabolism. GABA-T has been found to play a key role in the treatment of epilepsy with ATS and these findings deepen our understanding of how ATS intervenes in epilepsy. The identification of key metabolites and their associated pathways in this study offers potential novel therapeutic targets for epilepsy. By modulating these metabolites, future therapies could be designed to better manage the disorder. Moreover, the insights from network pharmacology can guide the development of more effective antiepileptic drugs, paving the way for improved clinical outcomes for patients.

## Figures and Tables

**Table 1:** Identified potential biomarkers regulated by ATS.

Metabolite	m/z	Formula	VIP	Trend (Model vs. Control)	Trend (ATS vs. Model)	Mode
D-erythro-1-(Imidazol-4-yl)glycerol 3-phosphate	273.0081	C_6_H_11_N_2_O_6_P	1.7857	up #	down *	−
Indolelactic acid	204.0666	C_11_H_11_NO_3_	1.7241	up #	down *	−
Leucocyanidin	341.0342	C_15_H_14_O_7_	2.2200	up #	down *	−
Ferulic acid	433.1150	C_10_H_10_O_4_	1.9900	up #	down *	−
10-Deoxymethymycin	474.2883	C_25_H_43_NO_6_	1.6613	down #	up *	−
3-(2-Furanyl)-2-propenal	123.0461	C_7_H_6_O_2_	1.8396	down #	up *	+
Piperonal	151.0397	C_8_H_6_O_3_	1.6126	down #	up *	+
Isobutyryl carnitine	232.1558	C_11_H_21_NO_4_	1.8639	down #	up *	+
(Carbamoylamino) (2R)-2,5-diaminopentanoate	155.0936	C_6_H_14_N_4_O_3_	2.4082	up #	down *	+
2-((3-Aminopyridin-2-yl)methylene)hydrazinecarbothioamide	413.1061	C_7_H_9_N_5_S	2.2088	up #	down *	+
Adenine	136.0625	C_5_H_5_N_5_	2.3440	up #	down *	+
2,5-Dimethyl-1H-pyrrole	96.0809	C_6_H_9_N	1.8603	up #	down *	+
3beta-[(Tetrahydro-2H-pyran-2-yl)oxy]androst-5-en-17beta-ol	357.2803	C_24_H_38_O_3_	2.2888	up #	down *	+
Epothilone C	477.2559	C_26_H_39_NO_5_S	2.1724	up #	down *	+
Gemcitabine	264.0811	C_9_H_11_F_2_N_3_O_4_	1.7711	down #	up *	+
Abscisic alcohol	251.1660	C_15_H_22_O_3_	1.9169	up #	down *	+
25-Hydroxyvitamin D3-26,23-lactone	446.3295	C_27_H_40_O_4_	1.8880	down #	up *	+
(2R,3R)-3-Methylornithinyl-N6-lysine	275.2032	C_12_H_26_N_4_O_3_	1.6326	up #	down *	+
1,2,3,4-Tetrahydroisoquinoline-3-carboxylic acid	177.0773	C_10_H_11_NO_2_	2.3825	up #	down *	+

# *p* < 0.05 vs. control group, * *p* < 0.05 vs. model group. Up indicates an increase, and down indicates a decrease in the model group vs. control group or ATS group vs. model group comparison.

## Data Availability

The original data are available upon reasonable request from the corresponding author.

## Abbreviations

The following abbreviations are used in this manuscript:

GABA-T	GABA transaminase
PTZ	Pentylenetetrazol
ATS	*Acorus tatarinowii* Schott
TCM	Traditional Chinese medicine
KEGG	Kyoto Encyclopedia of Genes and Genomes
GO	Gene Ontology
