# Correction: Chen et al. Metabolomics-Based Study on the Anticonvulsant Mechanism of *Acorus tatarinowii*: GABA Transaminase Inhibition Alleviates PTZ-Induced Epilepsy in Rats. *Metabolites* 2025, *15*, 175

**DOI:** 10.3390/metabo15050315

**Published:** 2025-05-08

**Authors:** Liang Chen, Jiaxin Li, Chengwei Fang, Jiepeng Wang

**Affiliations:** 1 School of Pharmacy, Guizhou University of Traditional Chinese Medicine, Guiyang 550025, China; chenliang029@gzy.edu.cn (L.C.); lijiaxin048@gzy.edu.cn (J.L.); maxiuping486@gzy.edu.cn (C.F.); 2 School of Basic Medical Sciences, Hebei University of Chinese Medicine, Shijiazhuang 050200, China

Error in Figure

In the original publication [1], there was a mistake in Figure 8. Figure 8 was placed incorrectly. The corrected Figure 8 appears below. The authors state that the scientific conclusions are unaffected. This correction was approved by the Academic Editor. The original publication has also been updated.

Text Correction

There were several errors in the original publication.

A correction has been made to the Abstract, as follows:

The value “57.9 µg/mL” should be “108.9 µg/mL”.

A correction has been made to the Results Section, as follows:

Results: Kyoto Encyclopedia of Genes and Genomes enrichment analysis revealed that ATS was involved in regulating multiple signaling pathways, mainly including the neuroactive ligand–receptor interaction and GABAerGamma-aminobutyrate transaminaseAminobu-tyrate Transaminaseapse signaling pathway. ATS treatment restored 19 metabolites in epiGamma-aminobutyrate transaminaseminobutyrate Transaminase rats, affecting lysine, histidine, and purine metabolism. GABA-T was found as a new key target for treating epilepsy with ATS. The IC_50_ of ATS for inhibiting GABA-T activity was 108.9 μg/mL. Through metabolomic analysis, we detected changes in the levels of certain metabolites related to the GABAergic system. These metabolite changes can be correlated with the targets and pathways predicted by network pharmacology. One of the limitations of this study is that the correlation analysis between altered metabolites and seizure severity remains unfinished, which restricts a more in-depth exploration of the underlying biological mechanisms. In the future, our research will focus on conducting a more in-depth exploration of the correlation analysis between altered metabolites and seizure severity.

Another correction has been made to Section 3.6, as follows:

*3.6. Inhibition Study of GABA-T*

ATS had a concentration-dependent inhibitory effect on GABA-T activity, as shown in Figure 8. The IC_50_ of ATS for inhibiting GABA-T activity was 108.9 μg/mL, which was lower than the reported value of vigabatrin (600 μg/mL) in the literature [21]. The results of the enzyme inhibition experiments indicated that the antiepileptic effect of ATS may be related to GABA-T, laying the foundation for further elucidating the antiepileptic mechanism of ATS.

The authors state that the scientific conclusions are unaffected. This correction was approved by the Academic Editor. The original publication has also been updated.

## Figures and Tables

**Figure 8:**
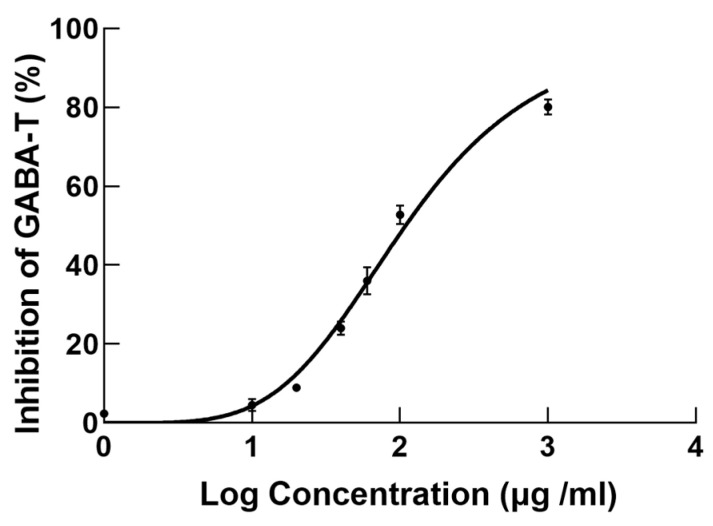
Inhibition curve of ATS on GABA-T (*n* = 3). Error bars indicate the standard error of the mean (SEM).
